# Transcatheter arterial embolization of cystic artery bleeding

**DOI:** 10.3389/fsurg.2023.1160149

**Published:** 2023-04-11

**Authors:** Hyo-Cheol Kim, Yun Soo Jeong, Kichang Han, Gyoung Min Kim

**Affiliations:** ^1^ Department of Radiology, Seoul National University Hospital, Seoul National University College of Medicine, Seoul, Republic of Korea; ^2^ Department of Radiology, Severance Hospital, Yonsei University College of Medicine, Seoul, Republic of Korea

**Keywords:** cystic artery, embolization, n-butyl cyanoacrylate, hemorrhagic cholecystitis, hemorrhage

## Abstract

**Purpose:**

The purpose of this study is to assess the safety and clinical outcomes of transcatheter arterial embolization (TAE) *via* the cystic artery for treating patients with bleeding from the cystic artery.

**Materials and Methods:**

This retrospective study included 20 patients who underwent TAE *via* the cystic artery between January 2010 and May 2022. Radiological images and clinical data were reviewed to evaluate causes of bleeding, procedure-related complications, and clinical outcomes. Technical success was defined as the disappearance of contrast media extravasation or pseudoaneurysm, as demonstrated on completion angiography. Clinical success was defined as discharge from the hospital without any bleeding-related issues.

**Results:**

Hemorrhagic cholecystitis (*n* = 10) was the most common cause of bleeding, followed by iatrogenic (*n* = 4), duodenal ulcer (*n* = 3), tumor (*n* = 2), and trauma (*n* = 1). Technical success was achieved in all cases, and clinical success was achieved in 70% (*n* = 14) of patients. Three patients developed ischemic cholecystitis as a complication. Six patients with clinical failure died within 45 days after embolization.

**Conclusion:**

TAE through the cystic artery has a high technical success rate in treating cystic artery bleeding, but clinical failure remains a common occurrence due to concurrent medical conditions and the development of ischemic cholecystitis.

## 1. Introduction

Transcatheter arterial embolization (TAE) is a fundamental procedure within the realm of interventional radiology. Several studies involving patients with gastrointestinal bleeding, hemoptysis, post-partum bleeding, and musculoskeletal bleeding have reported favorable outcomes with respect to the safety and effectiveness of TAE (1–5). Bleeding originating from the cystic artery may be associated with surgical cholecystectomy, hemorrhagic cholecystitis, gallbladder cancer, and prior percutaneous drainage of the gallbladder (6–9). Several case studies have documented the successful use of TAE in treating cystic artery bleeding (6–9), and embolization procedures through the cystic artery have been safely attempted for the management of hepatocellular carcinoma (10–12).

Nonetheless, there is insufficient literature available on the use of TAE *via* the cystic artery to treat patients who suffer from bleeding originating from this vessel. The purpose of this study was to report on the safety and clinical outcomes of TAE through the cystic artery for the treatment of patients with cystic artery bleeding.

## 2. Materials and methods

This retrospective study was approved by the institutional review board and the requirement for informed consent was waived. Between January 2010 and May 2022, 20 patients with bleeding from the cystic artery were treated with TAE *via* the cystic artery at two tertiary medical centers. The study population comprised 12 males and eight females [mean age, 64.2 ± 14.6 years (range, 34–85 years)].

The procedure for embolization *via* the cystic artery was conducted using a similar method to the previously described techniques (1–5). Access to the celiac trunk through the common femoral artery was achieved using a 5-Fr angiographic catheter. Digital subtraction angiography was performed using a 5-Fr catheter, followed by insertion of a 1.7–2.0 Fr microcatheter into the cystic artery. The embolic material used varied depending on the operator's decision.

A retrospective review of radiologic images and clinical data was conducted to identify causes of bleeding, procedure-related complications, and clinical outcomes. Technical success was defined as disappearance of contrast media extravasation or pseudoaneurysm demonstrated on completion angiography. Cessation of bleeding was defined as stabilization of hemoglobin level within 48 h of the embolization procedure, without the need for transfusion. Clinical success was defined as discharge from the hospital without any bleeding-related issues. Patients who met one of the following criteria were diagnosed with coagulopathy; prothrombin ratio > 1.5, partial thromboplastin time > 45 s, or platelet count of less than 80,000/㎕.

## 3. Results

20 patients (male, 12; female, 8) presented with hemoperitoneum (*n* = 4), hematemesis (*n* = 4), melena (*n* = 4), bloody fluid from percutaneous cholecystostomy tube (*n* = 4), abdominal pain (*n* = 2), hematochezia (*n* = 1), and jaundice (*n* = 1) (Table 1). The causes of bleeding from the cystic artery were attributed to hemorrhagic cholecystitis (*n* = 10), iatrogenic (*n* = 4), duodenal ulcer (*n* = 3), tumor (*n* = 2), and trauma (*n* = 1). The iatrogenic factors contributing to bleeding were percutaneous cholecystostomy (*n* = 2), surgical cholecystectomy (*n* = 1), and radiofrequency ablation (*n* = 1). Nine patients had coagulopathy and six patients had liver cirrhosis. Three patients had underlying cancers [hepatocellular carcinoma (*n* = 1), gallbladder cancer (*n* = 1), and gastrointestinal stromal tumor (*n* = 1)].

**Table 1:** Summary of clinical and radiological findings who underwent cystic artery bleeding embolization.

Patient No	Sex	Age	Underlying disease	Clinical manifestation	Cause of bleeding	Coagulopathy	ICU admission at presentation	Cholecystostomy prior to embolization	Embolized vessel	Embolic material	Complication	Clinical success	Subsequent treatment	Cause of death
1	Male	43	LC	Melena	Hemorrhagic cholecystitis	Present			Main	Blood clot	No	Yes	Surgical cholecystectomy	
2	Female	34	LC	Hemoperitoneum	Iatrogenic (cholecystostomy)	Present		Yes	Deep	Gelatin sponge	No	Yes		
3	Female	54	Duodenal GIST	Melena	Tumor bleeding				Main	NBCA	No	Yes		
4	Female	49	LC	Hematemesis	Hemorrhagic cholecystitis	Present			Main	NBCA	Ischemic cholecystitis	No		MOF, sepsis
5	Male	75	stroke	Hematochezia	Hemorrhagic cholecystitis	Present	Yes		Twig	NBCA	No	Yes		
6	Female	52	LC,	Hemoperitoneum	Iatrogenic (RFA)	Present			Twig	NBCA	No	Yes		
7	Female	71	gallbladder cancer	Jaundice	Tumor bleeding				Twig	NBCA	No	Yes		
8	Male	74	ESRD	Blood in cholecystostomy tube	Hemorrhagic cholecystitis		Yes	Yes	Twig	NBCA	No	No		MOF, sepsis
9	Male	48	Trauma	Hemoperitoneum	Trauma		Yes		Main	NBCA	Ischemic cholecystitis	Yes	Percutaneous cholecystostomy	
10	Male	48	DM, HTN, coronary disease	Blood in cholecystostomy tube	Hemorrhagic cholecystitis	Present		Yes	Main	NBCA	Ischemic cholecystitis	No		MOF, sepsis
11	Male	80		Hematemesis	Hemorrhagic cholecystitis				Deep	NBCA	No	Yes		
12	Female	78	HTN, atrial fibrillation, Pneumonia	Hematemesis	Hemorrhagic cholecystitis	Present	Yes		Deep	NBCA	No	No		pneumonia
13	Female	78	HTN, coronary disease	Hematemesis	Duodenal ulcer				Superficial	Coil	No	Yes	Surgical cholecystectomy	
14	Male	67	HTN, LC	Melena	Duodenal ulcer				Twig	NBCA	No	Yes		
15	Female	56	ESRD, duodenal perforation	Melena	Duodenal ulcer	Present	Yes		Main	Gelatin sponge	No	No		MOF, sepsis
16	Male	78	HTN, ESRD	Abdominal pain	Hemorrhagic cholecystitis				Deep	NBCA	No	Yes	Surgical cholecystectomy	
17	Male	64	Lung and kidney transplantation	Blood in cholecystostomy tube	Hemorrhagic cholecystitis			Yes	Deep	NBCA	No	Yes		
18	Male	80	LC, HCC, valve replacement	Hemoperitoneum	Hemorrhagic cholecystitis	Present	Yes		Deep	NBCA	No	No		MOF, sepsis
19	Male	85	HTN, DM, coronary disease, colon cancer	Blood in cholecystostomy tube	Iatrogenic (cholecystostomy)			Yes	Superficial	NBCA	No	Yes		
20	Male	70	Aortic disease, coronary disease	Abdominal pain	Iatrogenic (cholecystectomy)				Main	NBCA	No	Yes		

LC, liver cirrhosis; GIST, gastrointestinal stromal tumor; ESRD, end stage renal disease; DM, diabetes mellitus; HTN, hypertension; RFA, radiofrequency ablation; ICU, intensive care unit; NBCA, n-butyl cyanoacrylate; MOF, multiorgan failure.

Eleven patients presented to the hospital due to bleeding of the cystic artery, while nine patients were hospitalized for management of other medical problems. Six out of the nine hospitalized patients were admitted to the intensive care unit due to massive bleeding (*n* = 3), pneumonia (*n* = 1), heart failure (*n* = 1), and sepsis (*n* = 1) which were worsened by the development of cystic artery bleeding.

**Figure 1:**
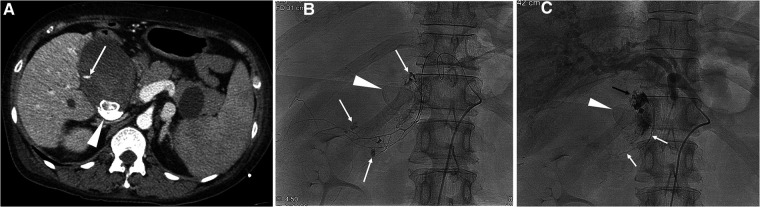
A 49-year-old female with liver cirrhosis and hemorrhagic cholecystitis. (**A**) CT scan shows extravasation of contrast media (arrow) in the gallbladder. Note gall stones (arrowhead). (**B**) Selective angiogram of the cystic artery shows multifocal extravasation (arrows) of contrast media. Note gall stones (arrowhead). A mixture of n-butyl cyanoacrylate and Lipiodol was used for embolization of the main cystic artery. (**C**) The final angiogram shows cessation of active bleeding. Note glue material in the cystic artery (white arrows) and in the lumen of the gallbladder (black arrow), and gall stones (arrowhead). Development of ischemic cholecystitis occurred after embolization of the cystic artery, resulting in sepsis and multiorgan failure. The patient died three days after cystic artery embolization.

**Figure 2:**
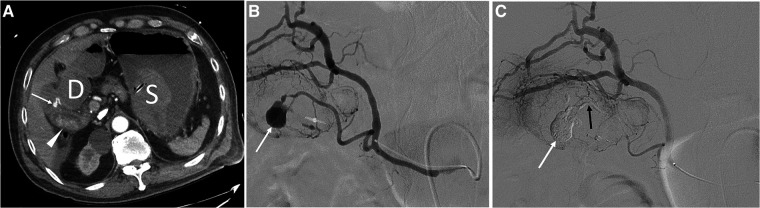
2 A 75-year-old male with hematochezia. (**A**) CT scan shows a pseudoaneurysm (arrow) located within the gallbladder. Note the presence of a blood clot with high attenuation in the stomach (S), duodenum (**D**), and gallbladder (arrowhead). (**B**) Right hepatic angiogram shows a pseudoaneurysm (arrow) from the superficial cystic artery. A mixture of n-butyl cyanoacrylate and Lipiodol was used for embolization of the bleeding point. (**C**) The final angiogram after embolization of the cystic artery shows a subtraction artifact of the pseudoaneurysm (white arrow) and superficial cystic artery (black arrow). Note intact perfusion of the deep cystic artery. The impacted gall stone was thought to induce development of a fistula between the gallbladder and duodenum, resulting in duodenal ulcer and hematochezia. The patient recovered without development of ischemic cholecystitis and was discharged two weeks after cystic artery embolization.

Embolization vessels were classified as the main cystic artery (*n* = 7) (Figure 1), deep cystic artery (*n* = 6), superficial cystic artery (*n* = 2) (Figure 2), and small bleeding twig (*n* = 5). Embolic materials used were n-butyl cyanoacrylate (NBCA) (*n* = 16), gelatin sponge particles (*n* = 2), coil (*n* = 1), and autologous blood clot (*n* = 1).

Technical success was achieved in all 20 (100%) patients. Cessation of bleeding was observed in 18 (90%) patients, and clinical success was achieved in 14 (70%) patients.

Median length of follow-up was 259 days (range, 2–3,669 days). Three patients who underwent embolization of the main cystic artery developed ischemic cholecystitis. Two patients with ischemic cholecystitis died due to sepsis and multiorgan failure resulting from ischemic cholecystitis. Four patients required subsequent treatment after cystic artery embolization, including one case of percutaneous cholecystostomy, and three cases of surgical cholecystectomy.

Six patients with clinical failure died in the hospital 3–45 days after embolization of the cystic artery. The causes of bleeding in these six patients were hemorrhagic cholecystitis (*n* = 5) and duodenal ulcer (*n* = 1). The causes of death were sepsis with multiorgan failure (*n* = 5) and pneumonia (*n* = 1). For these six patients, embolization vessels were classified as main cystic artery (*n* = 3), deep cystic artery (*n* = 2), and small bleeding twig (*n* = 1), and embolic materials used were NBCA (*n* = 5) and gelatin sponge particles (*n* = 1). Among the 11 patients who presented to the hospital due to bleeding of the cystic artery, clinical failure occurred in only one (9%) patient, while among the nine patients hospitalized for other medical problems, clinical failure occurred in five (56%) patients.

## 4. Discussion

In this study, hemorrhagic cholecystitis (50%, 10 out of 20) was the most common cause of cystic artery bleeding. Hemorrhagic cholecystitis is a progression of cholecystitis, which ranges from simple to complicated cholecystitis, hemorrhagic cholecystitis, gangrenous cholecystitis, and gallbladder perforation (13). Surgical cholecystectomy remains the gold standard option for treatment of hemorrhagic cholecystitis (14), but some patients may not suitable candidate due to poor general condition and anesthesia risks. In these cases, embolization of the cystic artery may be considered as an alternative procedure with a high rate of technical success.

In this study, the technical success rate was 100% and cessation of bleeding was achieved in 90% of patients. Liquid embolic material has the advantage of being able to cast the bleeding point itself, which is not affected by a patient's coagulopathy. However, there is a potential risk of ischemic cholecystitis when casting the entire cystic artery. Temporary embolic materials such as gelatin sponge particle or autologous blood can be used to reduce this risk, but there may be a high re-bleeding rate. Based on the findings of this small retrospective study, it is suggested that main cystic artery embolization with liquid embolic material may be associated with clinical failure despite technical success. When embolization of the main cystic artery is unavoidable on angiographic findings, embolization with temporary embolic material or surgical cholecystectomy should be considered as alternative options.

Despite the high technical success rate, the clinical success rate, defined as discharge from the hospital without bleeding-related problems, was only 70%. This result may be explained by two points. First, clinical failure occurred in five out of nine patients who were hospitalized for other medical problems, as the development of hemorrhagic cholecystitis in seriously ill patients may not be salvaged by TAE through the cystic artery. Second, embolization of the main cystic artery may be inevitable in cases of multifocal bleeding or extravasation/pseudoaneurysm involving the main cystic artery, and this can lead to ischemic cholecystitis and subsequent deterioration of the patient's general condition, potentially causing sepsis and multiorgan failure.

Duodenal ulceration with bleeding from cystic artery is an uncommon but possible condition, which has been reported in several case reports (15–17). An anterior or postbulbar ulcer can cause a massive gastrointestinal bleeding by ulceration into cystic artery (17), in case of adhesion between gallbladder and duodenum with chronic inflammation or ulceration. Duodenal ulcer was the cause of bleeding in three patients in this study. After endoscopic hemostasis failed in all three patients, TAE was performed and bleeding from cystic artery was confirmed by angiography.

Ischemic cholecystitis developed in three patients who underwent main cystic artery embolization. Considering their poor general condition, the referring physicians chose conservative management rather than surgical cholecystectomy, which resulted in two cases of mortality. Hence, surgical cholecystectomy should be considered as a life-saving procedure when ischemic cholecystitis occurs after TAE.

This study has several limitations. First, the study population was relatively small due to the uncommon nature of the disease entity, and the long study period. Thus, statistical analysis could not be performed to identify risk factors for clinical failure. Second, the use of liquid embolic material, which may have a high technical success rate and a relatively low clinical success rate, may deter some practitioners from using TAE through the cystic artery. However, the authors believe that use of liquid embolic material may be appropriate when superselective embolization is feasible. Third, in some cases, evaluation of ischemic cholecystitis after TAE through the cystic artery may be inaccurate or impossible, as many patients already had serious underlying disease. Fourth, the retrospective nature of the study leaves room for selection bias, especially in two tertiary hospitals known for their high patient volumes, where embolization procedures may have been overused due to their quick implementation.

In conclusion, TAE through the cystic artery has a high technical success rate in treating cystic artery bleeding, but clinical failure remains a common occurrence due to concurrent medical conditions and the development of ischemic cholecystitis.

## Data Availability

The original contributions presented in the study are included in the article/Supplementary Material, further inquiries can be directed to the corresponding author.
